# Assessment of airborne particles and bioaerosols concentrations in a waste recycling environment in Brazil

**DOI:** 10.1038/s41598-020-71787-0

**Published:** 2020-09-09

**Authors:** Caroline Fernanda Hei Wikuats, Eduardo Henrique Duarte, Kátia Valéria Marques Cardoso Prates, Laura Lahr Lourenço Janiaski, Bárbara de Oliveira Gabriel, Alex da Cunha Molina, Leila Droprinchinski Martins

**Affiliations:** 1 Federal University of Technology-Parana, 3131 Pioneiros Avenue, Londrina, PR 86036-370 Brazil; 2 Present Address: Brandon University, 270 18th Street, Brandon, MB R7A 6A9 Canada

**Keywords:** Air microbiology, Atmospheric science

## Abstract

This study aims to assess the concentrations of size-fractioned particle mass (PM_1.0_, PM_2.5_, PM_4.0_, PM_10_) and number (PNC_0.3_, PNC_0.5_, PNC_1.0_, PNC_2.5_), bacteria, and fungi in a Materials Recycling Facility (MRF) in Brazil. The measurements were performed inside the waste processing shed (P1) and in the outdoor environment (P2) during working days in winter and spring of 2017, and summer of 2019. A total of 2,400 min of PM, 1,440 min of PNC, and 216 samples of bioaerosols were collected in the morning and afternoon. P1 has the strongest air contamination with mean values of 475.5 ± 563.7 µg m^−3^ for PM_10_, 58.6 ± 36.0 cm^−3^ for PNC_0.3_, 1,088.8 ± 825.2 colony-forming units per cubic meter (CFU m^−3^) for bacteria, and 2,738.3 ± 1,381.3 CFU m^−3^ for fungi. The indoor/outdoor ratios indicated the large influence of indoor sources due to the activities performed inside P1 that promote the generation and resuspension of pollutants. Gram-positive bacteria dominated with 58.6% of indoor samples. Overall, our results show a critical indoor air quality situation in a Brazilian MRF, which may cause several health risks for waste pickers. Finally, we call attention to the lack of occupational exposure limits for bioaerosols in industrial workplaces and mainly in MRFs.

## Introduction

Urbanization, population growth, the development of large conurbations, and the modification in society's lifestyle have led to an increase in the generation rates of solid waste, which has been causing economic, environmental, and social problems worldwide^1–4^. Hence, its management turned into a very important activity for municipalities^5,6^ mainly due to environmental and public health reasons, as solid waste can pose risks to human health and contaminate the atmosphere, soil, and water resources^1,3,7^.

Developing countries have the worst municipal solid waste (MSW) management and are more severely impacted by improperly managed waste^8^. In Brazil, for example, around 79 million tonnes of solid waste are generated per year or 1.04 kg/capita/day, while the global average is 0.74 kg/capita/day^8,9^. Of this amount, 30–40% could be recycled and reused (excluding composting), however, only 13% is sent to recycling^10^.

The recycling segment is an important stage of solid waste management and it has advantages due to aggregation of economic value, contribution to the reduction of disposed of or incinerated waste, and the reuse of materials in industries^5,11^. Recycling generally occurs through an informal sector with several people in low- and middle-income countries working as waste pickers because of economic reasons^3,5,12–14^. In Brazil, around 800,000 people work as waste pickers in at least 802 recycling cooperatives according to data from the National Movement of Collectors of Recyclable Materials (MNCR)^15^ and the Business Commitment to Recycling (CEMPRE)^16^. In a Materials Recycling Facility (MRF) in Londrina, located in the southern region of Brazil, the monthly income of each waste picker is approximately US$ 235^17^, but this value generally varies according to the amount of waste processed and from one place to another.

Waste management includes activities in which exposure to hazardous chemical agents and airborne pollutants, such as particulate matter (PM) and bioaerosols, may be abundant and may trigger adverse effects on human health^18,19^. Particles of the respirable-size fraction (< 10 µm) are of major concern as they are small enough to penetrate the respiratory system^20–22^. PM exposure has been related to adverse health effects, such as respiratory symptoms, aggravated asthma, decreased lung function, increased hospital admissions, shortened life expectancy, and mortality^22–25^. It also can be potentially involved in cases of Alzheimer’s and Parkinson’s diseases^26^. Furthermore, exposure to bioaerosols may also cause cardiovascular and respiratory diseases, including asthma, pneumonia, bronchitis, and tuberculosis, in addition to allergic reactions (such as conjunctivitis and sinusitis) and organic dust toxic syndrome^27–30^.

The quality of the recyclable materials is reduced when the population do not properly separate the MSW in their households and, consequently, the air at MRFs may be contaminated during the processes performed (mainly during sorting and baling)^19,31,32^. As MSW can be easily colonized by bacteria and fungi, these microorganisms might aerosolize upon handling, i.e., turn into bioaerosols, and workers may be exposed to numerous biological agents, which pose health hazards^29,33,34^. It is reported that people who work handling recyclable waste may present an elevated risk of irritation of the skin, eyes, and throat, and gastrointestinal, respiratory, and musculoskeletal problems compared to those of other jobs^28,32,35^.

Although it is already known that the exposure to several biological agents is high and that there is a great risk of occupational health problems for workers of this sector^29,36^, the assessment of bioaerosols, particle mass (PM), and particle number concentrations (PNC) suspended in the air of materials recycling facilities is scarce. Most studies have been focusing on the people who collect the waste^33,37–40^ and on composting facilities and landfill sites^27,34,35,41–44^.

The PM and bioaerosols emissions at MRFs may vary due to design, operational conditions, types of materials processed, and meteorological variables^45–47^, singular for each facility studied. Thus, it is important to evaluate the presence of these airborne pollutants in this occupational setting to provide possibilities of controlling its air pollution through specific intervention plans based on demand. Furthermore, we used evaluation methodologies that can be easily applied to identify insalubrious environments and to provide the implementation of corrective actions for reducing the exposure and harmful effects on workers’ health in the MSW management sector, especially in low- and middle-income countries. In this direction, this study aims to assess the PM, PNC, bacteria, and fungi concentrations in a Brazilian recycling cooperative, which is a typical environment of the MSW management in the country.

## Results and discussion

### Particle concentrations analysis

**Figure 1:**
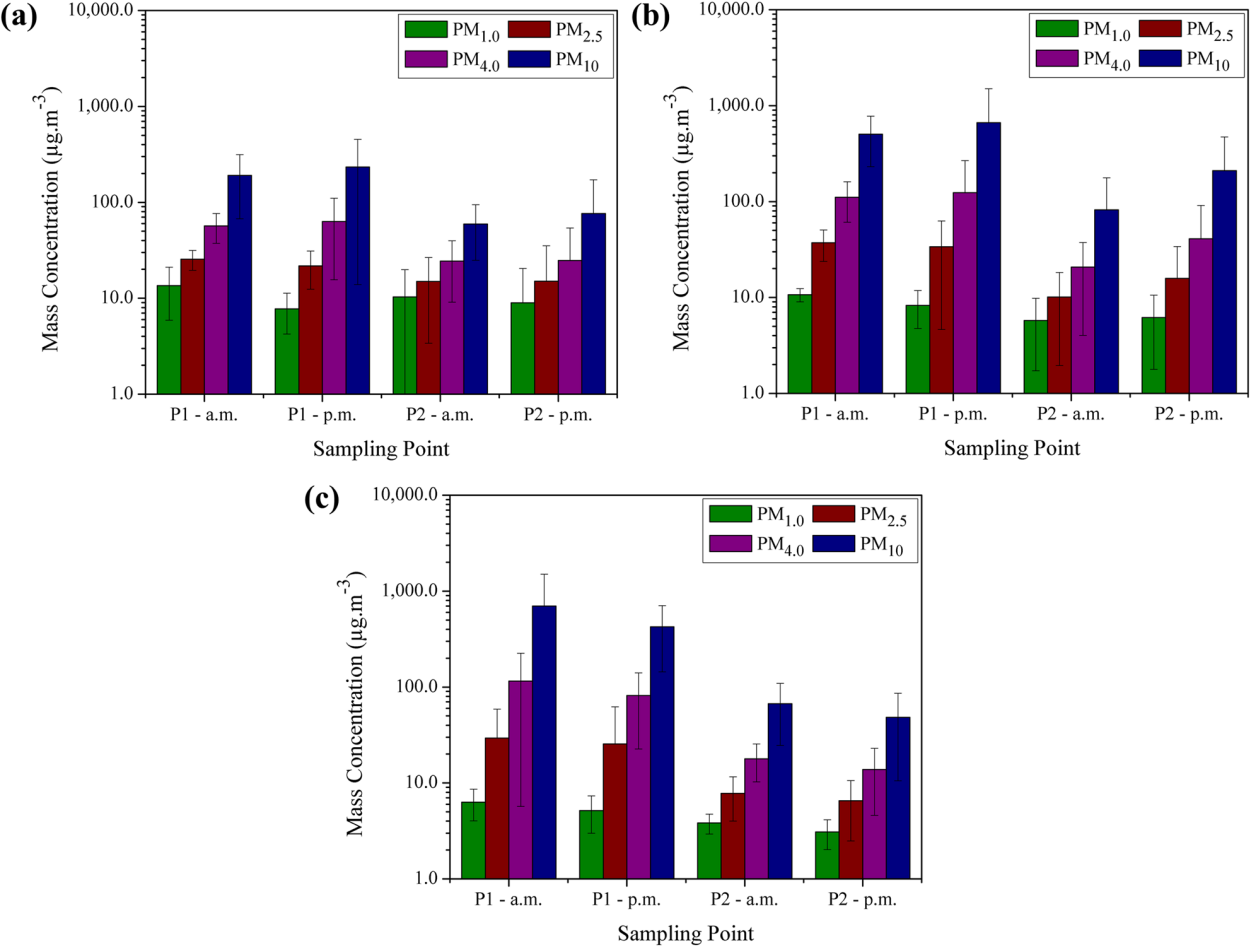
Mean mass concentrations (µg m^−3^) for all particle sizes measured during the morning and afternoon in winter (**a**), spring (**b**), and summer (**c**) in the waste processing shed (P1) and in the outdoor environment (P2).

Mean PM results in winter, spring, and summer during the morning and afternoon at each sampling point are presented in Fig. 1. The highest values of mass concentration, considering all four particle sizes sampled, were observed in P1 in the afternoon for winter and spring, and in P1 in the morning for summer (Fig. 1a–c). P1 represents the waste processing shed, a site without windows and mechanical ventilation, in which occurs the storage, sorting, and processing of the recyclable materials. The waste is separated into big polypropylene bags that are then moved through the floor (promoting the resuspension of PM) and sent to processing, which consists of baling the waste that is later stored until commercialization. Depending on the amount of sorted waste, the transfer of big bags with a forklift to the outdoor environment also occurred and they were stored until processing.

Moreover, it was observed the presence of trucks during the samplings (emission sources of PM) to unload the materials collected in the city. It is important to point out that variations in PM concentrations may be expected throughout the day (morning vs. afternoon), depending mainly on the waste characteristics and the tasks being performed. As expected, the presence of indoor sources of PM, such as resuspension, fuel combustion, storage, and sorting of the recyclable waste, in addition to scarce particle dispersion resulted in indoor concentrations generally higher than the outdoor concentrations. More detailed information regarding the activities observed during the sampling periods is presented in the Supplementary Table S1.

Higher concentrations of the largest particle diameters were observed (Fig. 1a–c), as expected and also reported by other authors^48,49^. This characteristic is particularly predominant for PM_4.0_ and PM_10_, with the latter ranging (in mean values) from 59.5 to 233.9 µg m^−3^ in winter, from 82.2 to 663.9 µg m^−3^ in spring, and from 48.4 to 699.3 µg m^−3^ in summer. Mean PM_2.5_ concentrations ranged from 15.0 to 25.5 µg m^−3^, from 10.1 to 37.2 µg m^−3^, and from 6.5 to 29.3 µg m^−3^ in winter, spring, and summer, respectively. Beal et al.^50^ found mean PM_2.5_ values of 4.4 µg m^−3^ during summer and 10.3 µg m^−3^ in winter for the city of Londrina.

Overall, comparing the seasons studied, the mean concentrations were mostly higher in winter for PM_1.0_ and in spring for PM_2.5_, PM_4.0_, and PM_10_, which was the season with the lowest contribution to the removal of PM through wet deposition. It had 39.2 mm of rain during the sampling week, compared to 58.4 mm in winter, and 68.2 mm in summer, according to the Agronomic Institute of Parana (IAPAR)^51^. Light breezes were observed in all seasons analysed and did not considerably influence the concentrations outdoors (P2). These meteorological data are presented in the Supplementary Table S2. Also, it was noted a larger amount of waste to be processed during the winter samplings, followed by summer and spring. The main operational conditions and activities were observed during the three seasons studied regarding unloading, sorting, and processing (Supplementary Table S1), but the shed was organized differently with more workstations for the separation of waste into the big bags in winter and spring, which may have contributed to the obtained concentrations of pollutants.

The results for PM presented non-normal distribution and we verified that the mean concentrations in winter, spring, and summer are statistically different (p = 0.05) applying the Kruskal–Wallis test. This behaviour reflects the large variability in indoor sources and activities, as well as the influence of outdoor atmospheric conditions on the indoor environment.

In Brazil, there are regulatory standards for occupational safety and health according to the Ministry of Labour’s Consolidation of Labour Laws (CLT), as amended by the Law no. 6,514/1977, which establishes insalubrious activities or operations based on tolerance limits for exposure to heat, cold, noise, humidity, non-ionizing radiation, chemical and biological agents, among others^52^. However, there are no quantitative guidelines for particulate matter (only for mineral dust, such as asbestos, manganese and its compounds, and crystalline silica). Therefore, we used the maximum recommended value (MRV) of 80 µg m^−3^ for total aerosols in indoor air established by the Brazilian Health Regulatory Agency’s Resolution no. 09/2003^53^. Concentrations obtained in this study were up to four, ten, and eleven times higher than the MRV in winter, spring, and summer, respectively, considering all four particle sizes sampled indoors. According to the World Health Organization (WHO)^54^, particulate matter from both indoor and outdoor sources is harmful to human health. Hence, the air quality guidelines recommended for PM (24-h mean: 50 µg m^−3^ for PM_10_ and 25 µg m^−3^ for PM_2.5_) can also be applied to indoor environments, which presented values several times higher than the standard for PM_10_ even taking into account that these guidelines are mean values for 24 h and the concentrations measured are for working hours.

The Brazilian regulation of 80 µg m^−3^ is not intended for indoor air at industrial environments or MSW management sites and occupational exposure limits for inhalable and total dust in the USA and Norway, for example, have been established as particulates not otherwise regulated, with guidelines of 10 mg m^−3^^55^. Hazard identification, risk assessment and control are used to define and describe hazards in the workplace and to implement control measures, such as the use of personal protective equipment (PPE) at different levels of protection considering, for example, the concentrations of indoor air contaminants in industrial occupational locations^56,57^. The values measured in the MRF indicate that workers must use masks to attenuate their exposure to airborne particles but the use of inappropriate PPE, as well as its incorrect use, was a common reality observed on-site during our measurements, besides administrative workers (office) who do not use PPE.

**Figure 2:**
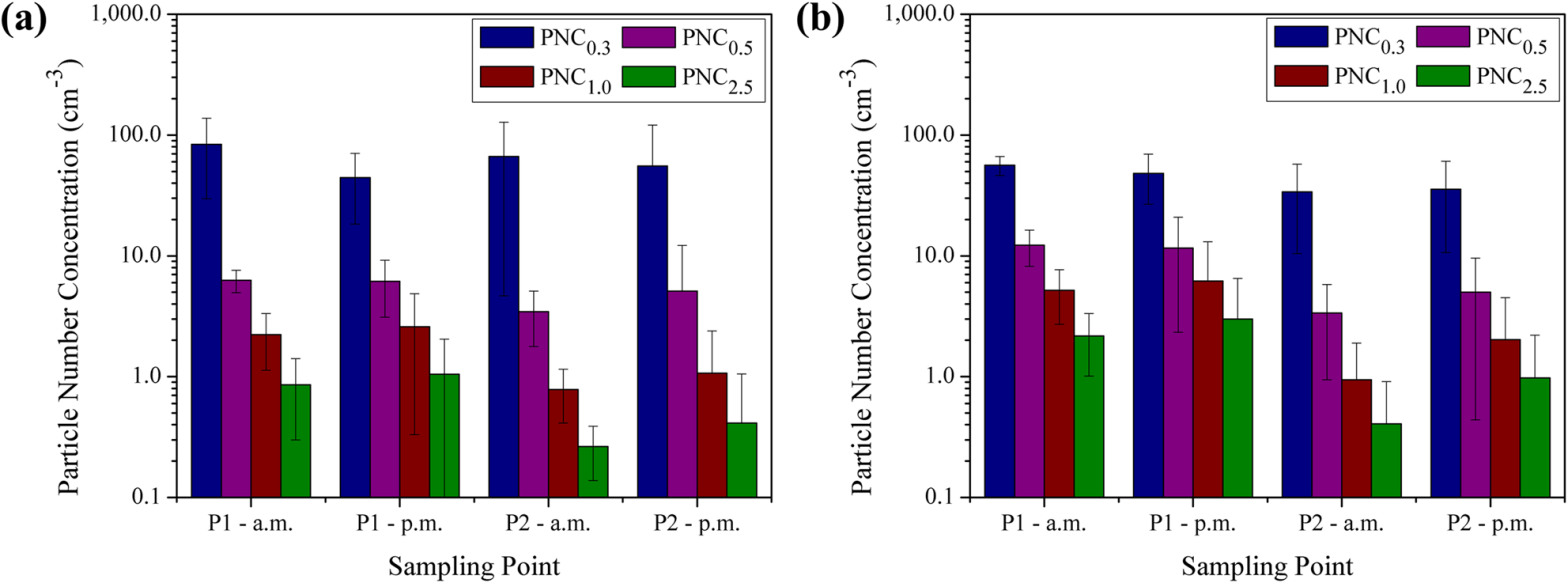
Mean number concentrations (cm^−3^) for all particle sizes measured during the morning and afternoon in winter (**a**) and spring (**b**) in the waste processing shed (P1) and in the outdoor environment (P2).

For the particle number concentrations (Fig. 2a,b), it was observed higher concentrations of the smallest sizes of particles measured, which is consistent and reported by other authors^34,58–60^. This tendency is prevalent for PNC_0.3_ that ranged from 44.5 to 83.7 cm^−3^ in winter and from 33.9 to 56.3 cm^−3^ in spring, in mean values.

Considering the four different particle sizes measured, the highest mean concentrations were recorded in P1 in the morning for winter and spring probably due to the accumulation of pollutants during the non-working hours when the shed remains closed and there is no air exchange. Also due to the frequency of activities being carried out, including the higher number of trucks arriving at the MRF to unload the waste in the morning compared to the afternoon. As well as for PM, the values for PNC presented non-normal distribution and the mean concentrations are statistically different at the 5% level of significance between winter and spring.

Overall, the values were mostly higher in winter for PNC_0.3_ and in spring for PNC_0.5_, PNC_1.0_, and PNC_2.5_. The smallest size fraction may present a greater potential in impairing human health than larger particles due to lung deposition and systemic spread in the human body, reaching the heart, brain, liver, and kidneys, among other organs^61–65^.

Beal et al.^50^ reported a decrease in the mass concentration of PM as higher values of relative humidity were registered due to the wet deposition of the pollutants, whereas higher temperature values caused an increase in the mass concentration of PM_2.5–10_. Total and respirable PM were negatively correlated with humidity and positively correlated with temperature^66^. Pateraki et al.^67^ also found a positive correlation between PM_10_, PM_2.5–10_, and temperature and a negative correlation for the pollutants and relative humidity. The same characteristics were observed in the present study for the mass concentration of PM (Fig. 1a,b) and the meteorological variables, except in summer, as this was the only season that rain was noted during one of the sampling days in the afternoon, which probably influenced the concentrations of PM between both periods studied. Tables with mean, median, and standard deviation values for the meteorological variables sampled outdoors at the Londrina weather station of the IAPAR and inside the waste processing shed are presented in the Supplementary Tables S2 and S3, respectively.

The mass concentration values of PM_2.5_, PM_10_, and dust measured in different MSW management facilities in the world, including this study, are shown in Table 1. The results are variable depending on the activity executed by the workers (driver or waste handler) and the characteristics of each location, such as the type of solid waste received, the season, and the process performed (collection or sorting). In most studies in solid waste facilities, PM exposure is estimated by dust mass concentration^28,29,36,46^. This research presents results in mass and number concentrations for PM by size fractions, assessing more detailed information related to air pollution in a MRF.

**Table 1:** Mean concentrations of PM_2.5_, PM_10_, and dust in municipal solid waste facilities around the world.

Location	Sampling site	Independent variables	PM_2.5_ (µg m^−3^)	PM_10_ (µg m^−3^)	Dust (µg m^−3^)	Reference
Canada	MSW recycling plant	Sorting				^28^
		Summer	–	–	1,100.0	
		Winter			400.0	
South Korea	MSW management plant	Collection and sorting				^29^
		Driver	–	–	600.0	
		Waste handler			900.0	
Finland	MSW management plant	Optic sorting process hall	–	–	1,200.0	^36^
		Optic sorting control room			300.0	
		Waste receiving plant			1,800.0	
United Kingdom	MRFs	Sorting	–	–	2,090.0	^46^
					3,600.0	
South Korea	MSW processing plant	Collection	73.3	300.2	–	^68^
		Sorting	61.6	458.1		
		Summer	93.1	247.3		
		Fall	145.1	384.0		
Portugal	Waste-sorting plants	WSP with mechanical ventilation	27.5	149.0	–	^69^
		WSP without mechanical ventilation	108.0	1,390.0		
Brazil	MRF	Sorting and processing				Present study
		Winter	23.7	211.8	–	
		Spring	35.5	584.5		
		Summer	27.4	562.4		

*MSW* municipal solid waste, *MRFs* materials recycling facilities, *WSP* waste-sorting plant, *PM* particulate matter, – not measured.

The PM_1.0_, PM_2.5_, PM_4.0_, and PM_10_ concentrations in this study are in the same range as the results found in other articles (Table 1). Despite the differences existing among the MRFs the highest values were observed predominantly during the sorting process^28,29,36,46,68^, and for people who directly handle the waste when evaluated the activity performed^29^, as well as noted in this research. Also, the absence of mechanical ventilation at the waste-sorting plant (WSP) in the study of Viegas et al.^69^ contributed to the mass concentration of PM_2.5_ and PM_10_. Thus, the particles may accumulate even more inside the waste shed analysed in our study due to the lack of a ventilation system and proper air exchange.

Regarding the season examined, our results diverge from those obtained by Lavoie and Guertin^28^, who found higher values in summer compared to winter, while Park et al.^68^ presented higher concentrations in fall when comparing to summer. In general, considering PM_2.5_ and PM_10_ in P1, the highest mean concentrations were registered in spring for the present study (Table 1), probably due to the more intense generation of PM from indoor sources and the infiltration of outdoor particles, as it was the season with the highest mean PM values in P2 and the lowest mean values of relative humidity (Supplementary Tables S2, S3), which probably caused a decrease in the pollutants removal via wet deposition. The amount of waste itself did not influence the concentrations obtained in spring, as it presented the lowest volume of waste to be processed compared to winter and summer. Therefore, the activities performed were substantial to the higher concentrations observed in spring.

Throughout the sampling week in August and February, respectively, 58.4 and 68.2 mm rainfall occurred compared to 39.2 mm in October^51^, which may have influenced the results since precipitation also contributes to the removal of atmospheric PM due to wet deposition^50,70,71^ (see Supplementary Tables S2, S3 that provide the data for all meteorological variables).

The indoor/outdoor (I/O) relationship was calculated for PM and PNC considering the samplings in the morning and afternoon in the three seasons studied (Table 2). The Brazilian Health Regulatory Agency’s Resolution no. 09/2003 recommended an I/O ratio lower than or equal to 1.5 for fungi, however, there is no specific ratio determined for PM. When the I/O ratio is higher than 1.5, a diagnosis of pollutant sources is necessary for a corrective intervention^53^.

**Table 2:** Indoor/outdoor ratios for PM and PNC in winter, spring, and summer.

Season	I/O		PM_1.0_	PM_2.5_	PM_4.0_	PM_10_	PNC_0.3_	PNC_0.5_	PNC_1.0_	PNC_2.5_
Winter	P1/P2	a.m	1.3	1.7	2.3	3.2	1.3	1.8	2.8	3.2
		p.m	0.9	1.4	2.5	3.1	0.8	1.2	2.4	2.5
Spring	P1/P2	a.m	1.9	3.7	5.3	6.1	1.7	3.6	5.5	5.3
		p.m	1.3	2.1	3.0	3.2	1.3	2.3	3.1	3.1
Summer	P1/P2	a.m	1.6	3.8	6.5	10.5	–	–	–	–
		p.m	1.7	3.9	5.9	8.8	–	–	–	–

– not measured.

The I/O ratios varied from 0.9 to 10.5 for PM depending on the season and period of the day analysed. Around 79% of the ratios were higher than 1.5, mainly for PM_4.0_ and PM_10_. During the morning samplings in spring and summer, the concentrations in P1 were much higher than in P2, resulting in values that were, respectively, four and seven times higher than 1.5 for PM_10_. Expressive values were also observed in the afternoon samples in summer as I/O ratios were four and six times higher than 1.5 for PM_4.0_ and PM_10_, respectively. This occurred probably due to the more intense generation of PM by indoor sources in spring and summer, although the infiltration of outdoor particles may also happen, whilst winter concentration values in P1 and P2 were considerably closer than in the other seasons. The mean concentration in P2 was higher in spring than in summer, probably due to the occurrence of 68.2 mm of rain during the sampling week in the latter season. Therefore, the ratios are generally higher in summer even though the mean concentrations are mostly higher in spring in P1.

For PNC, the values ranged from 0.8 to 5.5 and 75% of the ratios were higher than 1.5, mostly for PNC_0.5_, PNC_1.0_, and PNC_2.5_. As observed for mass concentration, the highest values were obtained in spring during the morning samplings, with ratios almost four times higher than 1.5 for PNC_1.0_ and PNC_2.5_.

In reviews regarding the relationship between indoor and outdoor PM, I/O ratios presented a wide range of values (tending to zero to over 10) due to the influence of distance to the sources, resuspension, type of ambient ventilation, air exchange rate, meteorological conditions, and the deposition velocity of particles^72,73^. Most studies presenting I/O data are performed in residential homes, commercial buildings, offices, schools, and universities and focus on measuring particle mass^73,74^. Thus, it is difficult to compare these environments to the occupational setting studied here, in addition to scarce information regarding PNC in indoor and outdoor air. Nevertheless, it is important to measure pollutants suspended in the indoor air to develop effective strategies to decrease human exposure and health risks associated with air pollution^74–76^.

### Bioaerosols analysis

**Figure 3:**
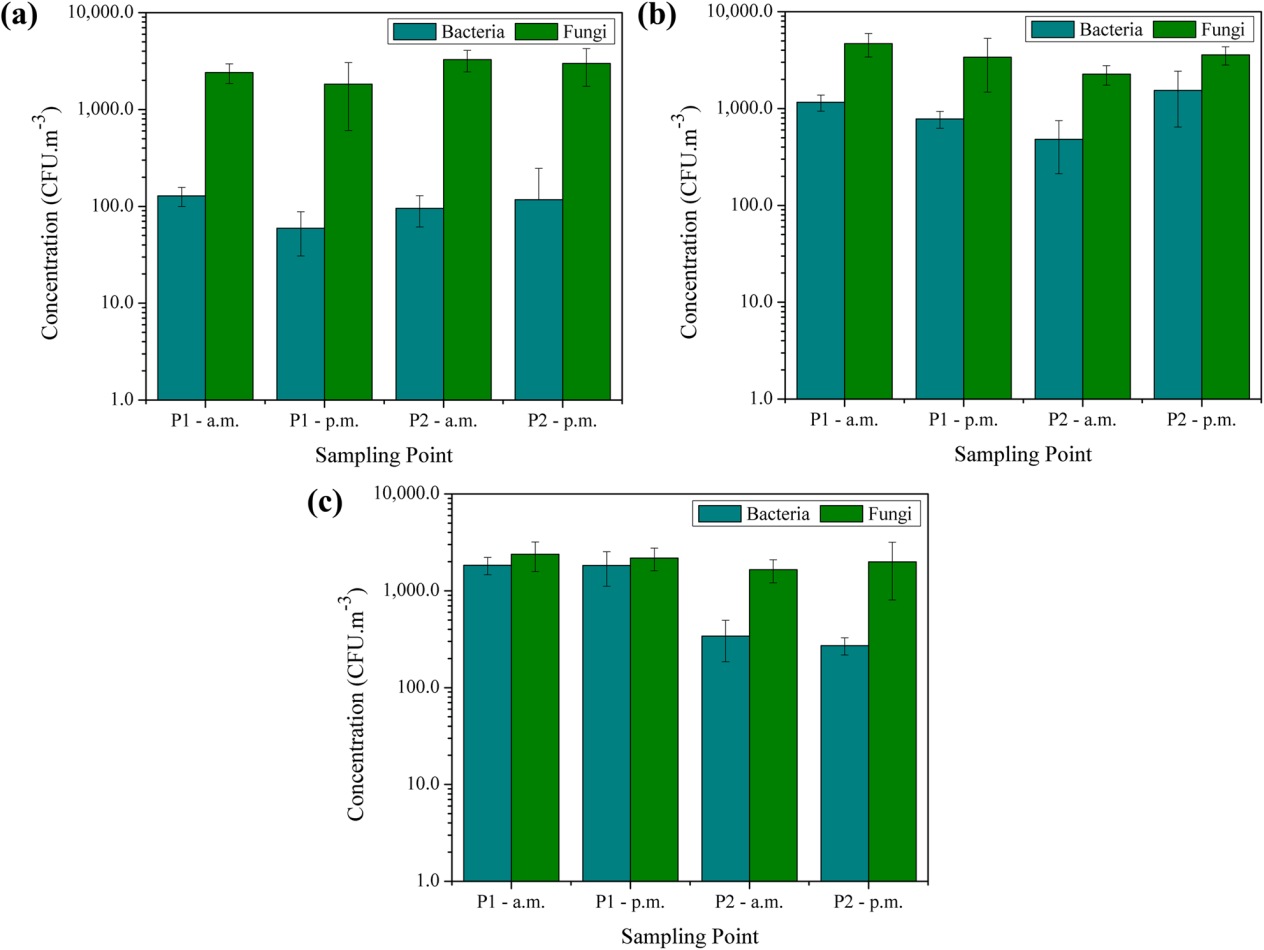
Mean concentrations of bacteria and fungi (CFU m^−3^) during the morning and afternoon in winter (**a**), spring (**b**), and summer (**c**) in the waste processing shed (P1) and in the outdoor environment (P2).

The mean concentrations of bacteria and fungi in the three seasons studied are shown in Fig. 3. The highest concentrations of bacteria were observed in P1 in the morning for winter and summer, and in P2 in the afternoon for spring. For fungi, this occurred in the morning in P2 for winter and in P1 for spring and summer. As aforementioned, P1 is the sampling point inside the waste processing shed, in which occurs activities related to the storage, sorting, and processing of the recyclable materials. On the other hand, P2 is the point located outdoors where there is an intense movement of trucks bringing the collected materials to the MRF. It was observed eventually the movement of other vehicles, such as cars and motorcycles, mainly from visitors. It also occurs the transfer of the big bags with sorted waste and the baled recyclable waste from the shed to the external environment using a forklift (promoting the resuspension of particles), which eventually becomes an open storage place and an emission source of airborne pollutants. Also, activities such as the storage and handling of the recyclable waste are considered as fundamental sources of bacteria and fungi at MRFs^28^.

The mean values ranged from 59.3 to 128.4 colony-forming units per cubic meter (CFU m^−3^) for bacteria and from 1,826.1 to 2,407.1 CFU m^−3^ for fungi in winter, while in spring the concentrations ranged from 480.4 to 1,537.6 CFU m^−3^ and from 2,261.8 to 4,689.6 CFU m^−3^ for bacteria and fungi, respectively. In summer, the mean concentration values were between 271.7 and 1,838.5 CFU m^−3^ for bacteria, and between 1,649.7 and 2,386.3 CFU m^−3^ for fungi. Given the environments analysed and the activities performed during the samplings, we expected higher concentrations for bioaerosols, but our results are probably underestimated because of the method used. Hence, the results obtained in this study for bioaerosols using the settle plate method may be lower than the actual concentrations that workers are inhaling.

Overall, the highest values were obtained in summer for bacteria and in spring for fungi (Fig. 3a–c) as warm seasons are more favourable to the growth and reproduction of these microorganisms. The data presented non-normal distribution and statistical differences were observed at the 5% level of significance comparing the mean concentrations in winter, spring, and summer when applying the Kruskal–Wallis test.

Threshold values for bioaerosols are not yet established by the WHO, the United States Environmental Protection Agency (U.S. EPA), and the American Conference of Governmental Industrial Hygienists (ACGIH), for example, due to limited data and variability in the possible health effects caused by the air microflora^30,77^. On the other hand, different governmental and private organizations have established quantitative guidelines without considering the effects on human health^77^.

The Brazilian Health Regulatory Agency’s Resolution no. 09/2003 determined an MRV of 750 CFU m^−3^ for fungi in indoor air^53^. In Portugal, the MRV was reported in the Technical Note NT-SCE-02 of the National System for Energy and Indoor Air Quality Certification of Buildings as 500 CFU m^−3^ for bacteria and fungi^78^. In Denmark and some other Scandinavian countries, guidelines were established for total bacteria and Gram-negative bacteria as 10,000 and 1,000 CFU m^−3^, respectively, for an 8-h period^28^. Except for the Scandinavian countries, it is noteworthy that these guidelines were not established specifically for indoor air at MSW management sites, but we used these as reference values in the absence of more suitable ones.

Analysing the indoor setting in our study and the Brazilian Resolution, the concentration values of fungi in P1 were around three times higher than 750 CFU m^−3^ in winter and summer. In spring, the values were up to six times higher than the established MRV. Comparing with the Portuguese reference value, concentrations of fungi were almost five times higher than 500 CFU m^−3^ in P1 in winter and summer, and nine times higher than the MRV in spring. For bacteria, this was observed in spring and summer, when they were two and almost four times higher than the mentioned value, respectively. On the other hand, none of the concentrations exceeded the limit established in Denmark for total bacteria. As the Brazilian and Portuguese MRVs are aimed for indoor settings for public use with air-conditioning and/or buildings, we can assume that the values sampled in the MRF do not correspond to the activities carried out that generate airborne microorganisms due to the use of the settle plate technique. Thereby, although these results are not as high, the Occupational Safety and Health Administration (OSHA) of the United States related that 1,000 CFU m^−3^ indicates air contamination but not necessarily unsafe or hazardous conditions, which depend on the type of airborne microorganisms^79^.

Unfortunately, we only have this vague guideline in Brazil that urgently should be improved and specific regulations for waste pickers should be implemented due to the possible hazards of occupational exposure to bioaerosols. As stated in the regulatory standards of the Brazilian Ministry of Labour^52^, the biological agents are characterized qualitatively according to the activity performed, such as people who work in permanent contact with patients in isolation due to infectious diseases, wastewater, and urban waste (collection and industrialization). Thus, working with MSW is considered insalubrious, but no quantitative guidelines were established for waste pickers' health and safety in Brazil.

Meteorological variables, such as temperature and relative humidity, can affect the presence of bioaerosols in the air^80^. Dungan^80^ also related that airborne microorganism’s viability declines when temperature rises and relative humidity decreases. Frankel et al.^81^ found significant correlations between temperature, relative humidity, and the concentrations of bacteria and fungi, and so did Ren et al.^82^ for fungi in indoor air and Gamero et al.^83^ for fungi at a landfill site. Temperature and relative humidity, respectively, were negatively and positively correlated with bacterial and fungal concentrations^84^. On the other hand, Goh et al.^85^ related that indoor temperature and relative humidity were constant during their samplings, so these variables did not present any substantial impact on the concentrations of indoor fungi and bacteria. While the results in the literature are different, we found higher indoors (P1) concentrations of bacteria and fungi in the morning, when the temperature was lower (negative correlation), and humidity was higher (positive correlation), similar to Dungan^80^ and Green et al.^84^ (see Supplementary Table S3 for the meteorological data measured inside the waste processing shed). In P2, wind speed did not influence the bioaerosols concentrations (Supplementary Table S2).

The concentration values of bioaerosols, as total bacteria, Gram-negative bacteria, and fungi, measured in different MSW management facilities in the world, including this study, are shown in Table 3. Great variability in the parameters analysed and the results obtained in other studies is observed. The sampling methods were also different, and each sampling site has its characteristics regarding the type of solid waste received and the meteorological variables of the location.

**Table 3:** Mean concentrations of bioaerosols in municipal solid waste facilities in the world.

Location	Sampling site	Independent variables	Total Bacteria (10^3^ CFU m^−3^)	GNB (10^3^ CFU m^−3^)	Fungi (10^3^ CFU m^−3^)	Reference
Canada	MSW recycling plant	Sorting				^28^
		Summer	21.9	0.5	19.2	
		Winter	6.1	N.D.	4.9	
South Korea	MSW management plant	Collection and sorting				^29^
		Driver	18.0	7.2	8.7	
		Waste handler	220.0	81.0	24.0	
Finland	MSW management plant	Optic sorting process hall	68.9	–	220.0	^36^
		Optic sorting control room	3.0		6.1	
		Waste receiving plant	1.2		3.5	
South Korea	MSW management plant	Collection	11.0	12.0	3.7	^86^
		Sorting	31.0	32.0	43.0	
		Summer	18.0	22.0	21.0	
		Fall	8.2	4.2	3.3	
Poland	MSW management plant	Sorting				^87^
		Sorter	65.0	–	102.0	
		Machine operator	29.0		126.0	
Brazil	MRF	Sorting and processing				Present study
		Winter	0.09	–	2.1	
		Spring	1.0		4.0	
		Summer	1.8		2.3	

*MSW* municipal solid waste, *MRF* materials recycling facility, *CFU* colony-forming unit, *GNB* gram-negative bacteria, *N.D*. not detected, – not measured.

The highest concentrations of bioaerosols were registered mainly during the sorting process^28,29,36,86,87^ and for the samplings performed in summer^28,86^. Regarding the season studied, Lavoie and Guertin^28^ claim that the concentration values of microorganisms measured during winter are always lower than during summer. In the present research, a similar result was observed as bacteria and fungi concentrations were lower in the winter compared to the summer. However, the highest average concentration for bacteria was registered in summer, while for fungi this occurred in spring (Table 3). Concentrations of fungi generally increase in spring and peak in summer^88,89^, although results are diverse in the literature. Černá et al.^90^ had higher fungi concentrations in spring in comparison to winter, while Nadal et al.^91^ presented higher values of bioaerosols in summer. When analysing different environments than the ones studied in this research, Gonçalves et al.^92^ found higher values at the beginning of spring in São Paulo, while Emygdio et al.^93^ presented a higher number of total spores in spring, but different genera had a large variability regarding seasonal characterization.

In general, the values measured in other studies presented in Table 3 are from 10^2^ to 10^5^ CFU m^−3^, while we obtained values ranging from 10^1^ to 10^3^ CFU m^−3^. The lack of a sampling protocol makes it difficult to compare the results since authors used different sampling methods, such as multi-stage impactors for the collection of airborne microorganisms on agar plates and air sampling pumps to measure the bioaerosols on polycarbonate or fibreglass filters. We used the settle plate method, which is a gravitational and passive (non-volumetric) sampling procedure used to enable the gathering of particles by the gravitational force on agar plates^77^ and the results are reproducible, reliable, and portray real conditions of the site studied^94,95^. Also, this technique is economically feasible, easily applicable, and allows samplings from different places at the same time without the disturbance of airflow^94,95^. However, it has limitations regarding the collection of smaller particles because the dry deposition of particles is a function of their mass, as well as the conversion of the number of CFUs counted on the agar plates in CFUs per cubic meter of air, presenting underestimated results compared to active sampling methods.

In Table 4 we present the I/O ratios that were calculated for bacteria and fungi concentrations in the morning and afternoon for the three seasons studied. The recommended I/O ratio for fungi is equal to or lower than 1.5 and there is no specific value for bacteria^53^. The values ranged from 0.5 to 6.7 for bacteria and from 0.6 to 2.1 for fungi. Four values, which represent 33.3%, were higher than 1.5 in spring and summer. This is an indication of the influence of activities performed inside the shed on levels of both bacteria and fungi observed indoors. Thus, higher indoor concentrations may be triggered by indoor bioaerosol sources^96^, but the infiltration of outdoor particles may also happen.

**Table 4:** Indoor/outdoor ratios for bacteria and fungi in winter, spring, and summer.

Season	I/O		Bacteria	Fungi
Winter	P1/P2	a.m	1.4	0.7
		p.m	0.5	0.6
Spring	P1/P2	a.m	2.4	2.1
		p.m	0.5	1.0
Summer	P1/P2	a.m	5.4	1.4
		p.m	6.7	1.1

In the present research, only four values were higher than 1.5, so the outdoor contribution to the concentration of airborne microorganisms is expressive probably due to the storage of recyclable items outside the waste shed at the MRF studied. Pictures of the outside environment (P2) are presented in the Supplementary Material. We did not find I/O ratios in similar environments (management of MSW), therefore no comparisons were made with other studies.

Although the results for bioaerosols are not as high as in other studies given the underestimation caused by the settle plate method, we observed a great diversity of bacterial and fungal colonies with different colours, shapes, margins, and textures through the macroscopic morphological characterization, which suggests genus and species richness in the air of the MRF. The results from the Gram staining technique showed that Gram-positive bacteria dominated the indoor samples of isolated colonies with 58.6%. Observing their microscopic morphology, the colonies of bacteria were then grouped into Gram-positive bacilli, Gram-positive cocci, Gram-negative bacilli, and Gram-negative cocci, which represented 46.6, 12.1, 24.1, and 17.2%, respectively.

Gram-positive bacteria also have been found as the most abundant airborne bacteria when analysing indoor samples in previous studies^96–99^. Airborne Gram-negative bacteria present brief periods of survival^96^, hence their comparatively small presence in the air of the MRF studied. Nevertheless, both Gram-positive and Gram-negative bacteria present cell wall components with pro-inflammatory agents, which may cause health problems such as respiratory symptoms^18^.

## Conclusions

Bioaerosols, PM, and PNC concentrations were assessed in the environment of a Brazilian MRF, which is how the recyclable waste is managed in the country. The strongest air contamination by these pollutants was observed inside the waste processing shed (P1) due to the variety of activities that promote the generation and resuspension of pollutants. Mean values in P1 were 475.5 ± 563.7 µg m^−3^ for PM_10_, 58.6 ± 36.0 cm^−3^ for PNC_0.3_, 1,088.8 ± 825.2 CFU m^−3^ for bacteria, and 2,738.3 ± 1,381.3 CFU m^−3^ for fungi, considering all the seasons and periods of the day analysed.

The concentrations were predominantly higher in summer for bacteria and spring for fungi, PM, and PNC. The I/O ratios demonstrated the large influence of indoor sources on indoor concentrations, mainly for PM and PNC, which presented 79 and 75% of values higher than 1.5, respectively, during the three seasons studied.

Temperature and relative humidity may have influenced the behaviour found for these pollutants as notable correlations were observed among these parameters and the concentrations of PM and bioaerosols. Also, the operational conditions carried out at the MRF that result in airborne particles and microorganisms are responsible for this great variability of results. The studied MRF certainly has several genera and species of bacteria and fungi suspended in the air, which should be elucidated in future studies.

Our results show a critical indoor air quality situation with considerable concentration values mainly for particulate matter, which may cause several health risks for the waste pickers. Although we used an evaluation technique with easy application and economic feasibility, the bioaerosols results are most likely underestimated because of the passive sampling procedure. In Brazil, there is a lack of studies regarding the working environments and conditions at MRFs and the occupational health risks that waste pickers face daily. Hence, we worked toward a start to fill the gap of this knowledge. The temporal characteristics of particulate matter and bioaerosols emissions should be further explored given the high variability of activities occurring at the MRF during the samplings. The association between the exposure to these pollutants and the occurrence of occupational health problems also could be investigated in future studies.

It is suggested that the waste pickers use PPE, mainly gloves and masks, to mitigate the exposure to PM, PNC, and bioaerosols. We also recommend a detailed hazard identification, risk assessment and control to implement specific intervention plans based on the requirements of the MRF, such as in which areas and/or activities the use of PPE should be intensified, the real need of a ventilation system for the dispersion of pollutants, among other issues. For example, having enough doors and windows at MSW management sites is an economic and potential measure to improve air exchange naturally. Nevertheless, regulations for waste workers’ health and safety, in addition to design and operational conditions at MRFs should be implemented in Brazil considering the possible risks of occupational exposure to airborne pollutants and the opportunity of increasing natural ventilation. Moreover, it is imperative to have a better inspection and bigger investments in the infrastructure of MRFs to enhance the working settings for this important economic class and to minimize the occurrence of health problems, which generate high costs for the public health system.

## Materials and methods

### Study area characterization

A Materials Recycling Facility (MRF) located in Londrina, in the southern region of Brazil (23° 18′ 54″ S; 51° 12′ 52″ W), was used for the study. Londrina has an estimated population of 569,733 inhabitants according to the Brazilian Institute of Geography and Statistics (IBGE)^100^ and is a typical medium-sized city in Brazil. Furthermore, there is a daily production of 465 tonnes of municipal solid waste^101^, representing approximately 0.80 kg/capita/day.

**Figure 4:**
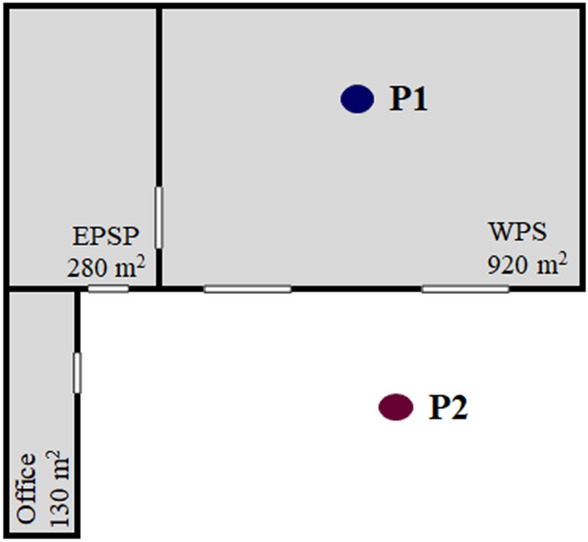
Sketch of the sampling points in the waste processing shed (P1) and in the outdoor environment (P2). *EPSP* expanded polystyrene processing, *WPS* waste processing shed.

The MRF is in the western region of Londrina, an urbanized area that has many residences, small industrial activities, and intense flow of light- and heavy-duty vehicles due to the proximity of a state highway. Furthermore, there is the movement of the trucks at the MRF itself that are used in the waste collection. The constructed area of the MRF is around 1,330 m^2^ represented by a shed and an office for its management. Moreover, there is an outdoor space where the processed waste is stored. The shed has a total area of 1,200 m^2^ even though the waste is stored, sorted, and processed by the waste pickers in an area of 920 m^2^, while the remaining 280 m^2^ are reserved for the processing of expanded polystyrene (Styrofoam) (Fig. 4). It has two large doors where the trucks enter to unload the recyclable waste, but it has no windows. Hence, two sampling points were selected for this study: the first inside the waste processing shed (P1) and the second in the outdoor environment (P2) (Fig. 4). We included pictures of the MRF as a Supplementary Material to provide a better view of the sampling sites (Supplementary Figs. S1, S2).

The MRF studied is responsible for the collection of recyclable waste from approximately 90,000 households, which represents 39% of its total number in Londrina according to the Municipal Transit and Urbanization Company (CMTU)^102^. The collection is carried out with trucks from Monday to Friday in different areas determined by the CMTU, and then the recyclable waste is taken to the MRF for its sorting. The trucks enter the waste processing shed for the unloading of the materials to the floor. In 2017, workers used to have static screening tables to sort the materials in multiple workstations. In 2019, the MRF installed a conveyor to move the materials with two workstations along it. However, not all MRFs in Brazil have conveyors to separate the recyclable waste. The waste sorting process occurs manually by 26 workers, who put the materials in big polypropylene bags (90 × 90 × 110 cm with a loading weight of 1.5 tonnes) considering the following categories: paper, cardboard, metal, aluminium cans, plastic bags, PET bottles, Tetra Pak packages, Styrofoam, and residual waste. An average of 86.7 tonnes of recyclable waste is commercialized per month in the waste processing shed studied.

### Sampling design and instrumentation

A total of 2,400 min of PM, 1,440 min of PNC, and 216 samples of bioaerosols were collected during working days in August and October of 2017, and in February of 2019, in the morning and afternoon. These months represent the respective seasons in Brazil: winter, spring, and summer. In every sampling campaign, 12 measurements were performed for PM and PNC of 60 min each, except for summer in which we carried out 16 measurements of PM (60 min each) and none for PNC due to operational obstacles. For bioaerosols, 72 samples were collected in every one of the seasons for 10 min. The data were clustered into morning and afternoon of each season studied.

Air temperature and relative humidity were measured using the Onset HOBO sensor (Model UX100-023, Onset Computer Corporation, USA), simultaneously with PM, PNC, and bioaerosols in the waste processing shed (P1). All measurements were performed around 1.2 m from the ground and the sampling apparatuses were placed in each environment as indicated in Fig. 4. Meteorological variables from the Agronomic Institute of Parana (Londrina weather station: 23° 21′ 16″ S; 51° 09′ 51″ W) were compiled to represent the conditions of the atmosphere outdoors during the sampling periods (see Supplementary Material).

PM and PNC measurements were carried out during 60 min each at the two sampling points simultaneously, with a flow rate of 2.83 L min^−1^ and 1 min of temporal resolution, using the Mass Particle Monitor (Model Aerocet 831, Met One Instruments, USA) and the Particle Counter Monitor (Model 804, Met One Instruments, USA). The first one was used for the measurement of the PM mass concentrations considering the sizes of 1.0 (PM_1.0_), 2.5 (PM_2.5_), 4.0 (PM_4.0_), and 10 μm (PM_10_) in diameter. The second one was used to measure the PNC for 0.3 (PNC_0.3_), 0.5 (PNC_0.5_), 1.0 (PNC_1.0_), and 2.5 μm (PNC_2.5_) in diameter.

### Bioaerosols assessment

The settle plate method was used to collect bioaerosols samples (in triplicate) in Petri dishes containing Plate Count Agar (PCA), for the cultivation of bacteria, and Sabouraud Dextrose Agar (SDA), for the cultivation of fungi. Sampling was performed concomitantly with PM and PNC measurements in each of the three seasons studied and the exposure time of the agar plates to the air was 10 min^53,103^ at both collection points. In each experiment, we included contamination control plates that were not exposed to the air and all of them resulted in culture negative.

A new bioaerosols collection was performed in September of 2019 using the passive and active sampling procedures simultaneously to calculate their concentrations (in CFU m^−3^) since the equations found in the literature for the settle plate method using the colony-forming units (CFUs) presented different results^94,104,105^. Therefore, we established a relationship based on the results obtained from both sampling techniques to calculate the bioaerosols concentrations for the previous passive samplings.

The settle plate method sampling was done as aforementioned. For the active sampling, we used the MAS-100 NT equipment (MBV AG, Switzerland) in which bioaerosol collection occurs through the impaction process, i.e., the microorganisms can impact directly on the agar plate. The flow rate was 100 L min^−1^ resulting in 250 L of air collected in 2.5 min. The culture media utilized were Tryptic Soy Broth (TSB) for bacterial cultivation and Dichloran Rose Bengal Chloramphenicol (DRBC) for fungal cultivation. DRBC medium plates were also used for the settle plate sampling to evaluate a possible interference of the culture medium in the CFU count, which was not observed.

After sampling, agar plates were incubated at 35 °C ± 1 °C for 24 h for bacteria and at room temperature (± 25 °C) for 96 h for fungi^106^. Subsequently, the CFUs were counted in each Petri dish and we established ratios (7.4 for bacteria and 31.1 for fungi) between the concentrations obtained by the active sampling in CFU m^−3^ and the CFU values of the settle plate method. Then, the concentrations in CFU m^−3^ of the previous passive samplings were calculated, representing a correction since the growth of microorganisms on the agar plates is lower when using the settle plate method.

Bacterial colony morphology was observed through its form, elevation, and margin according to Rodina^107^ and then the colonies were isolated according to their macroscopic characteristics. Gram staining technique was applied for 58 isolated colonies of bacteria following the methodology described by Benson^106^ and microscopic morphological analysis was carried out observing the shape of cells: spheres (cocci), rods (bacilli), and spirals. Colony diversity was also analysed for fungi observing its main macroscopic aspects^108^.

## Supplementary information

Supplementary file1
