# Supplementary material for: Assessment of airborne particles and bioaerosols concentrations in a waste recycling environment in Brazil
Source: Sci Rep. 2020 Sep 9;10:14812. doi: 10.1038/s41598-020-71787-0 (PMC7481203; doi:10.1038/s41598-020-71787-0)
Supplement: Supplementary file 1 — Supplementary file1 [file 41598_2020_71787_MOESM1_ESM.pdf]

Supplementary Material

Assessment of airborne particles and bioaerosols concentrations in a waste recycling environment in Brazil

Caroline Fernanda Hei Wikuats[1,*], Eduardo Henrique Duarte[1], Kátia Valéria Marques Cardoso Prates[1], Laura Lahr Lourenço Janiaski[1, 2], Bárbara de Oliveira Gabriel[1], Alex da Cunha Molina[1], Leila Droprinchinski Martins[1]

[1] Federal University of Technology – Parana, 3131 Pioneiros Avenue, Londrina, PR, 86036-370, Brazil

[2] Present Address: Brandon University, 270 18th Street, Brandon, MB, R7A 6A9, Canada

*** Corresponding Author**: Federal University of Technology – Parana, 3131 Pioneiros Avenue, Londrina, PR, 86036-370, Brazil. Phone number: +55 (43) 3323-1133, e-mail address: carolinewikuats@alunos.utfpr.edu.br

Supplementary Figure S1 Waste processing shed of the MRF.

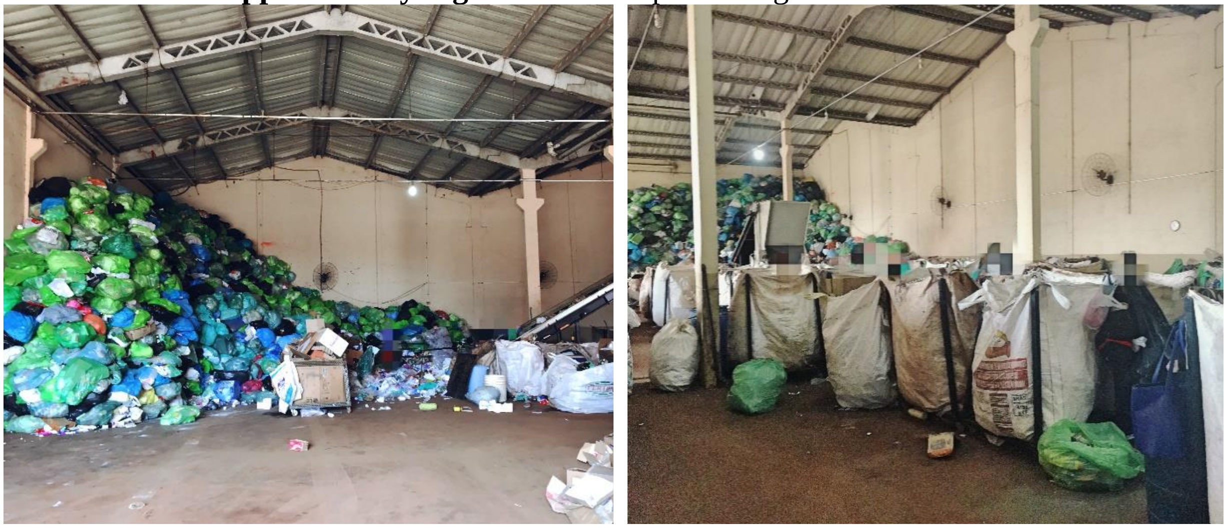

Supplementary Figure S2 Outdoor environment of the MRF.

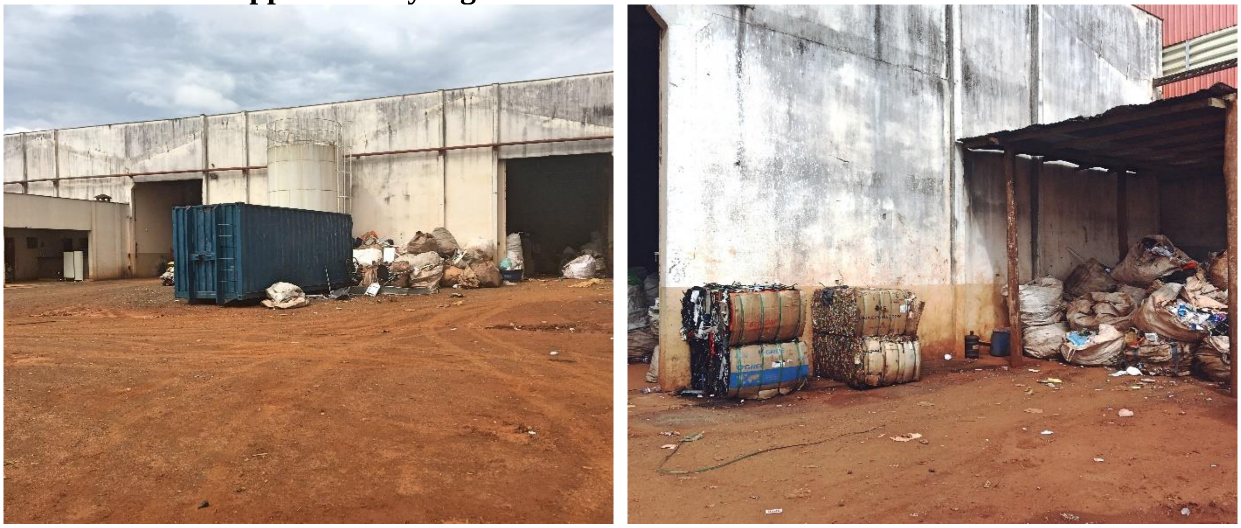

Supplementary Table S1 Information on the activities observed during the sampling periods at the MRF.

Activities	Winter	Spring	Summer
Unloading of the recyclable waste with trucks inside the WPS[a]	X	X	XX
Storage of the materials inside the WPS until sorting	XX	X	XX
Manual sorting of the recyclable waste placed on screening tables/conveyor	XX	XX	XX
Separation of the waste into big bags	XX	XX	XX
Movement of some big bags through the floor to the processing area	XX	XX	XX
Transfer of some big bags to the outside with a forklift	XX	XX	
Storage of some big bags outdoors until processing	XX	XX	
Waste processing via baling	XX	XX	XX
Transfer of the baled waste to the outdoor environment with a forklift	XX	XX	XX
Storage of the baled waste outdoors until commercialization	XX	XX	X
Sweeping the floor inside the WPS	X		XX
Trucks manoeuvring in the exterior site	X	X	X
Shattering glass outside the WPS			X

[a] WPS: waste processing shed; X: less frequently; XX: more frequently.

Supplementary Table S2 Descriptive statistics for the meteorological variables in winter, spring, and summer measured outdoors*.

Meteorological Variables		Winter		Spring		Summer	
		a.m.	p.m.	a.m.	p.m.	a.m.	p.m.
**Temperature (°C)**	Mean	16.2	20.3	20.3	26.8	25.0	27.2
	Median	16.0	19.5	20.1	26.1	25.5	28.0
	Std. Dev.[a]	2.4	4.4	4.5	4.8	2.3	2.6
**Relative Humidity (%)**	Mean	83.4	63.4	58.8	35.8	81.3	69.1
	Median	92.2	65.3	57.0	34.7	80.6	66.0
	Std. Dev.	18.2	36.8	17.6	6.3	9.8	13.0
**Wind speed (m/s)**	Mean	2.2	2.2	3.0	3.3	1.7	2.9
	Median	2.2	2.3	2.7	3.5	1.5	2.9
	Std. Dev.	0.6	0.6	1.0	0.7	0.7	1.0
**Cumulative Rainfall (mm)**		58.4		39.2		68.2	

* Data from the Agronomic Institute of Parana for the Londrina weather station; [a] Std. Dev.: standard deviation.

Supplementary Table S3 Descriptive statistics for the meteorological variables in winter, spring, and summer measured in the waste processing shed (P1).

**Meteorological Variables**		**Winter**		**Spring**		**Summer**	
		a.m.	p.m.	a.m.	p.m.	a.m.	p.m.
**Temperature (°C)**	Mean	20.7	23.1	23.7	27.1	29.0	30.9
	Median	20.3	23.5	23.4	27.1	29.0	29.8
	Std. Dev.[a]	0.9	1.1	1.5	1.0	1.2	2.7
**Relative Humidity (%)**	Mean	68.5	57.2	57.9	44.7	61.0	53.8
	Median	69.6	57.9	58.5	44.6	62.0	55.0
	Std. Dev.	2.7	2.9	4.9	1.6	5.4	7.2

[a] Std. Dev.: standard deviation.
